# An AI-driven multi-objective framework for optimizing window dimensions considering energy demand and thermal comfort

**DOI:** 10.1038/s41598-026-39795-8

**Published:** 2026-02-12

**Authors:** Seyed Mohammad Reza Adel Nasab, Hossein Rabiei

**Affiliations:** 1 Faculty of Architecture and Urbanism, Bauhaus University, Weimar, Germany; 2 Faculty of Physics and mathematics, Leibniz University, Hannover, Germany

**Keywords:** Window design, Multi-objective optimization, Thermal comfort, Energy efficiency, Surrogate modeling, Hot-dry climate, Energy science and technology, Engineering, Mathematics and computing

## Abstract

Designing energy-efficient windows requires balancing energy demand and occupant thermal comfort, particularly during early-stage design. This study presents an artificial intelligence–driven multi-objective framework for optimizing window dimensions in a simplified office building under Tehran’s hot-dry climate. A parametric simulation model was developed using Grasshopper, Ladybug Tools, and EnergyPlus to generate a dataset covering variations in window width and height. For each configuration, annual cooling energy demand (kWh), annual heating energy demand (kWh), and adaptive thermal comfort, defined as the percentage of occupied hours within the ASHRAE 55 adaptive comfort range, were evaluated. An artificial neural network surrogate model was trained using decorrelated geometric inputs obtained through principal component analysis, achieving high predictive accuracy. The surrogate was embedded within a multi-objective evolutionary optimization process to explore trade-offs between energy demand and thermal comfort. The resulting Pareto-optimal solutions indicate that intermediate window sizes provide a balanced compromise between reduced energy demand and improved comfort. The proposed framework supports performance-informed window design decisions at the early design stage while remaining extensible to more complex design scenarios.

## Introduction

### Background

Designing energy-efficient building envelopes remains a central challenge in architectural and environmental design, particularly in climates characterized by strong seasonal contrasts^1^. Among envelope components, windows play a critical role in regulating heat exchange, solar gains, and indoor thermal conditions. While larger window areas can improve daylight availability and passive solar heating during colder periods, they often increase cooling energy demand and overheating risk in warm seasons. Conversely, smaller windows may reduce cooling demand but compromise indoor comfort and winter energy performance. These opposing effects make window design a fundamentally multi-objective problem, requiring a careful balance between energy demand and occupant thermal comfort from the early stages of design.

Recent years have seen a surge in research marrying artificial intelligence (AI) techniques with multi-objective optimization (MOO) to enhance building energy performance. Classic evolutionary algorithms like the Non-dominated Sorting Genetic Algorithm II (NSGA-II) have long been applied to building design problems^2–4^. However, contemporary studies increasingly adopt advanced many-objective algorithms such as NSGA-III, especially to handle complex design scenarios involving numerous conflicting objectives^5^. By around 2015, NSGA-III began appearing in building performance optimization applications, demonstrating superior capability over NSGA-II in managing larger objective sets^5–8^. For example, early uses of NSGA-III addressed multi-objective energy management (balancing energy cost, emissions, and comfort) in smart buildings, outperforming other evolutionary approaches^9^. Likewise, Kim et al. applied NSGA-III in building retrofit planning to jointly optimize energy efficiency, greenhouse gas emissions, and cost^10^. These advances underscore a trend: genetic algorithms remain central for exploring design trade-offs, but newer variants like NSGA-III are increasingly favored for their improved convergence and diversity handling^4,11,12^.

Parallel to algorithmic improvements, the scope of optimization objectives has broadened. Early MOO studies mostly minimized energy use and cost, but from 2017 onward there is a marked inclusion of occupant-centric metrics such as thermal comfort and indoor environmental quality^13–16^. It is now common to see studies simultaneously targeting energy consumption, thermal comfort, visual (daylight) performance, and sometimes indoor air quality (IAQ) or life-cycle cost.

### State of the art

Recent advances in building performance simulation and optimization have enabled systematic exploration of window design alternatives using multi-objective approaches. Previous studies have commonly employed evolutionary algorithms to optimize window-related parameters, focusing primarily on reducing annual energy consumption while maintaining acceptable indoor thermal conditions. In many cases, thermal comfort has been incorporated as an additional objective alongside energy-related indicators, allowing designers to investigate trade-offs between energy efficiency and occupant well-being. To mitigate the computational burden associated with simulation-driven optimization, surrogate modeling techniques such as artificial neural networks have increasingly been adopted to approximate building performance and accelerate the search process. These approaches have demonstrated strong potential for supporting performance-informed design decisions, particularly during early design stages when geometric parameters remain flexible. Moreover, Recent studies have increasingly emphasized model interpretability and robustness in AI-driven building performance optimization. For instance, Zhou et al. employed interpretable ensemble learning to assess indoor visual comfort, enabling clearer attribution of design variables to performance outcomes. Similarly, Yuan et al.^17,18^integrated explainable machine learning with multi-objective optimization to investigate the relationship between architectural spatial forms and thermal comfort in courtyard buildings. Other recent works have focused on calibrating and improving the reliability of thermal comfort indices using ensemble learning approaches and real-world data^19–22^. These advances highlight a growing shift toward transparent and interpretable AI frameworks in building performance research. Table 1 summarizes representative studies on window-related energy and thermal comfort optimization, highlighting their objectives, methods, and key limitations in relation to the present study.

**Table 1:** Major studies contributions to the field.

Title	Authors	Year	Design variables	Research tools	Optimization method	Building performance
Deep Reinforcement Learning for Building HVAC Control	^23^	2017	HVAC control actions	Simulation + DRL	Deep Q-Learning	HVAC energy, comfort
Multi-objective optimization for sustainable building design	^24^	2017	Insulation, HVAC	EnergyPlus + GA	NSGA-II	Energy, discomfort
Multi-Objective Optimization of the Envelope of Building with Natural Ventilation	^25^	2018	Window size, orientation	EnergyPlus	NSGA-II	Heating/cooling balance
Passive design optimization of newly-built residential buildings in Shanghai	^26^	2018	Envelope features	EnergyPlus + ANN	NSGA-II	Cooling/heating, CTR
Multi-objective optimization of building energy performance and indoor thermal comfort by combining ANN and metaheuristic algorithms	^27^	2021	Insulation, orientation	TRNSYS + ANN	NSGA-II / MOPSO	Load and PMV improvement
Optimizing Thermal Comfort and Energy Use for Learning Environments	^28^	2021	Set-point temps	EnergyPlus	NSGA-II	Energy use, discomfort hours
Optimization of classroom design in hot-humid climate	^29^	2021	Classroom layout	EnergyPlus	NSGA-II	Cooling, comfort
Thermal performance optimization of envelope in retrofits	^30^	2021	Envelope U-values	Analytical modeling	Gradient-based	LCC, energy savings
Machine learning-based method for detached energy-saving residential form generation	^31^	2022	Geometry/massing	Parametric + ML	ML-guided generation	Annual energy demand
Improving CO₂ and occupants’ thermal comfort in a residential building using GA	^32^	2023	Ventilation rates	CONTAM + EnergyPlus	GA	CO₂, comfort
Energy Efficiency in HVAC Systems with ML and MPC	^33^	2023	HVAC operation	Real data + RBFNN	MPC + RBFNN	COP, energy
AI model for defining indoor thermal comfort in UK homes	^34^	2024	Thermostat, occupancy	ASHRAE DB + ANN	ANN comfort controller	Comfort coverage
Simulation-based multi-objective optimization in hot climates	^35^	2024	Retrofit envelope	EnergyPlus	NSGA-II	Energy savings, discomfort
BIM and AI for Energy Performance in Riyadh and Dubai	^36^	2024	Envelope & window specs	BIM + ML	Feature-based ML	EUI reduction
Low computational cost retrofits using ML surrogates	^37^	2024	Retrofit options	EnergyPlus + surrogate	NSGA-III + ML	Retrofitting cost vs. comfort
BIM + XAI + MOO for green building energy optimization	^38^	2024	Insulation, glazing	LightGBM + BIM	NSGA-II + BO	Energy, CO₂, comfort
ML and GA for climate-adaptive energy and comfort optimization	^39^	2024	WWR, shading, set-points	EnergyPlus + XGBoost	Bayesian + NSGA-II	Robustness, overheating
ML-based predictive model for comfort and energy optimization in smart buildings	^40^	2024	Set-point profiles	Sensor + ML	Ensemble ML	PMV, HVAC savings

### Research gap

Despite significant progress in simulation-based and AI-assisted window design optimization, several gaps remain in the current body of research. Existing studies predominantly emphasize annual energy performance indicators, while the systematic and interpretable examination of trade-offs between energy demand and thermal comfort has received comparatively less attention. In particular, although thermal comfort is often included as an optimization objective, its interaction with energy performance is frequently reported in an aggregated or opaque manner, limiting its usefulness for early-stage design reasoning.

Moreover, many surrogate-assisted optimization frameworks rely on complex machine-learning models that function largely as black boxes, offering limited insight into how basic geometric design parameters influence performance outcomes. This lack of transparency reduces their practical value for architects and designers who require not only optimal solutions but also an intuitive understanding of design–performance relationships during early decision-making phases. Additionally, previous studies often investigate a broad range of design variables or complex building configurations, leaving a gap in focused, methodologically clear frameworks that examine fundamental window dimension parameters in a controlled and interpretable setting. Addressing these gaps is essential to support performance-informed window sizing decisions that balance energy demand and thermal comfort at the early design stage.

### Research objectives and contributions

#### Research objectives

Based on the identified research gaps, the primary objective of this study is to develop a transparent and computationally efficient framework for exploring the trade-offs between window-related energy demand and thermal comfort at the early design stage. To achieve this overarching goal, the specific objectives of the study are as follows:

- To generate a parametric simulation dataset that captures the influence of window width and height on annual cooling energy demand, annual heating energy demand, and adaptive thermal comfort in a simplified office building.
- To develop and validate a surrogate modeling approach capable of accurately approximating building performance outcomes while significantly reducing computational cost.
- To apply a multi-objective optimization process to identify Pareto-optimal window dimension configurations and systematically quantify energy–comfort trade-offs.
- To analyze the resulting solution space in order to provide interpretable insights into how variations in window size affect energy demand and thermal comfort under a hot-dry climate context.

#### Research contributions

The main contributions of this study can be summarized as follows:

- This work proposes a structured AI-assisted framework that integrates parametric simulation, surrogate modeling, and multi-objective optimization to support early-stage window dimension design.
- The study provides a clear and interpretable assessment of trade-offs between annual energy demand and adaptive thermal comfort, addressing limitations associated with black-box optimization approaches.
- By focusing on fundamental window dimension parameters within a controlled modeling environment, the proposed framework offers practical insights that are directly applicable to early-stage architectural decision-making.
- The results contribute empirical evidence on the energy–comfort implications of window sizing in a hot-dry climate, serving as a methodological reference for future studies incorporating additional design variables or comfort dimensions.

## Materials and methods

### Case study description

**Fig. 1:**
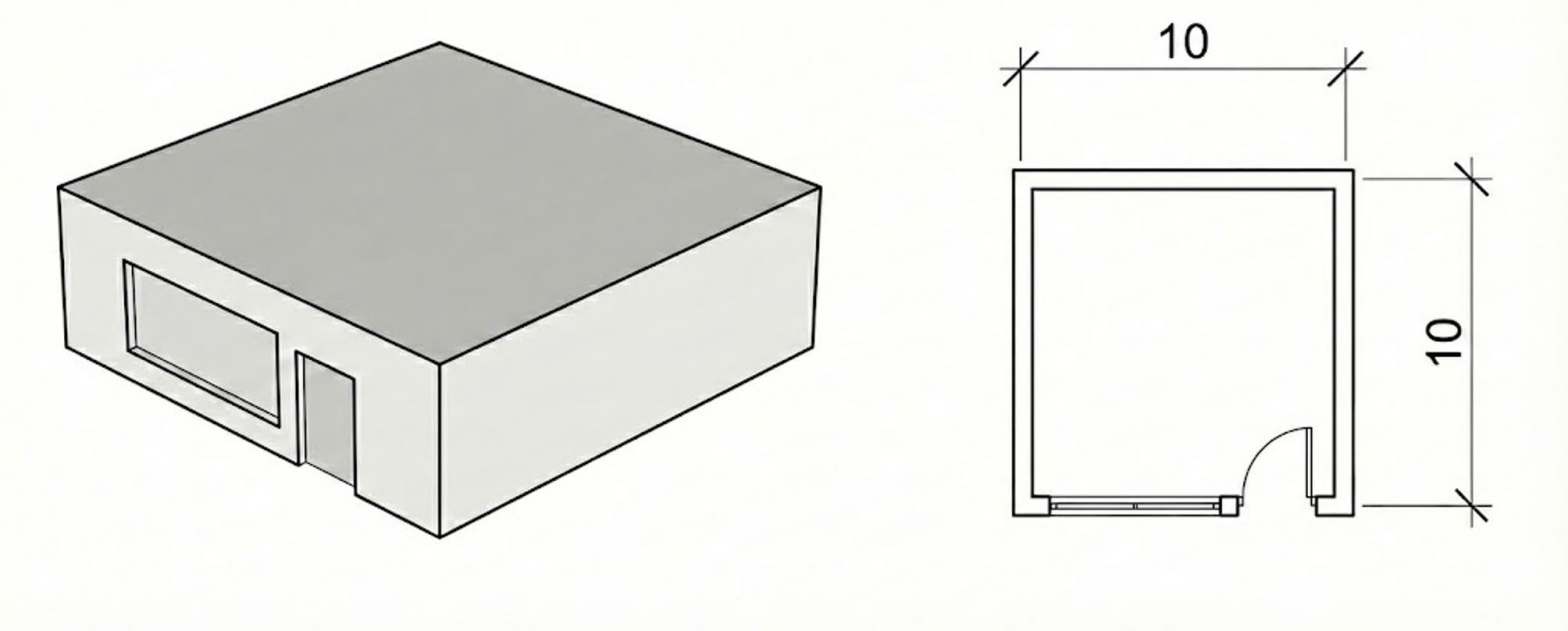
Case-study shoebox office model.

The case study is a one-story office building located in Tehran, Iran, modeled as a simplified shoebox geometry (Fig. 1) to ensure transparency and control over boundary conditions. The building has a fixed rectangular footprint and orientation, while a single window located on the main façade is parametrically defined. Window width and window height are selected as the primary design variables and systematically varied to investigate their influence on building energy demand and thermal comfort performance.

### Simulation environment and dataset generation

**Fig. 2:**
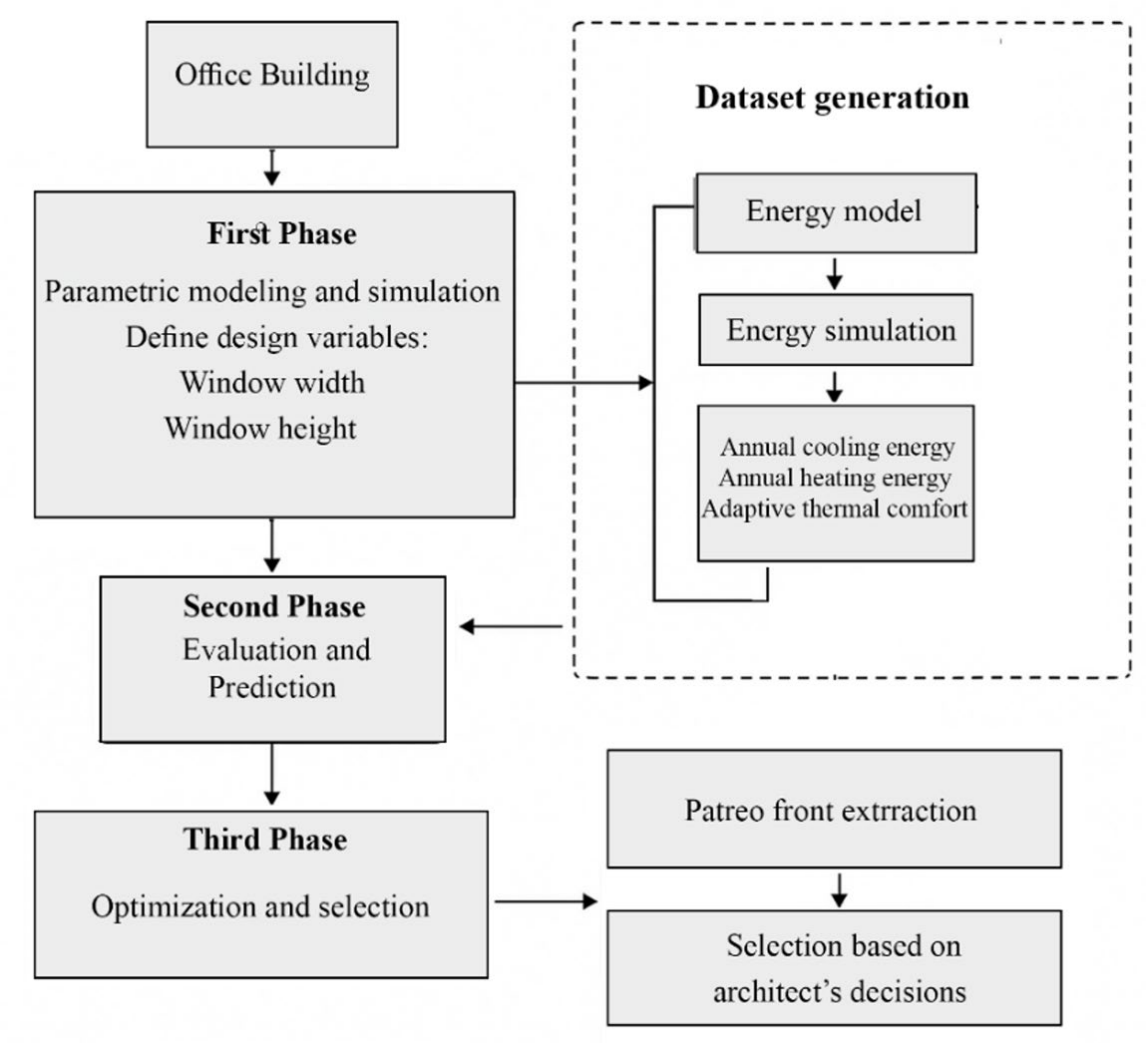
Overview of the proposed three-phase framework, including parametric simulation, surrogate-based prediction, and multi-objective optimization for window dimension design.

As can be seen in Fig. 2, a parametric simulation framework was developed using Grasshopper with Ladybug Tools and Honeybee, integrated with the Colibri parametric iterator. Climatic conditions were represented using the EnergyPlus Weather (EPW) file for Tehran. Window width and height were varied within a predefined range of 0.5 to 3.5 m, covering both moderate and large window configurations relevant to early-stage façade design. For each configuration, building performance was evaluated using EnergyPlus simulations to obtain:

- Annual cooling energy demand (kWh),
- Annual heating energy demand (kWh),
- Adaptive thermal comfort, expressed as the percentage of occupied hours within the ASHRAE 55 adaptive comfort range.

Annual heating and cooling energy demands were evaluated under a mechanically conditioned scenario with fixed thermostat setpoints, consistent with standard EnergyPlus energy-performance assessments. In contrast, thermal comfort was assessed using an adaptive comfort index under a free-running operational assumption, reflecting naturally ventilated conditions as defined in ASHRAE Standard 55. This separation was adopted because adaptive comfort models are not applicable to fully air-conditioned operation. Consequently, energy and comfort metrics were not evaluated under an identical operational mode, and the comfort index should be interpreted as a relative indicator of passive thermal acceptability rather than a direct measure of comfort under conditioned operation.

#### Boundary conditions

All simulations were conducted under fixed boundary conditions to isolate the influence of window geometry on building energy demand and thermal comfort. Envelope properties, internal gains, HVAC settings, occupancy schedules, and building orientation were kept constant across all simulation runs. This approach ensures that variations in annual cooling energy demand, annual heating energy demand, and adaptive thermal comfort can be attributed solely to changes in window width and height. The fixed boundary conditions applied in the EnergyPlus model are summarized in Table 2. For reference, a single EnergyPlus simulation of the shoebox office model required approximately 50–65 s on the test machine (Intel i7 CPU, 32 GB RAM), whereas surrogate inference required less than 0.01 s per evaluation. As a result, the full optimization process could be completed within minutes rather than days, supporting the suitability of the proposed framework for early-stage design exploration.

**Table 2:** Fixed simulation boundary conditions used in the energyplus model.

Category	Parameter	Value	Notes
Geometry	Number of floors	1	Shoebox office model
Geometry	Floor-to-floor height	3.0 m	Typical office
Orientation	Facade orientation	South-facing	Fixed
Envelope	External wall U-value	0.45 W/m²·K	Iranian building code
Envelope	Roof U-value	0.30 W/m²·K	Iranian building code
Envelope	Floor U-value	0.40 W/m²·K	Assumed
Glazing	Window U-value	2.8 W/m²·K	Double glazing
Glazing	SHGC	0.55	Manufacturer data
Infiltration	Air change rate	0.5 ACH	Typical office
HVAC	System type	Ideal Loads Air System	EnergyPlus
HVAC	Cooling setpoint	26 °C	Fixed
HVAC	Heating setpoint	20 °C	Fixed
Comfort	Comfort model	ASHRAE 55 adaptive	Free-running
Weather	Weather file	Tehran EPW	EnergyPlus database

### Data cleaning and preprocessing

Prior to surrogate model training, the simulation dataset was screened to remove invalid and inconsistent records. Specifically, simulation runs were excluded if any of the output variables (annual cooling energy demand, annual heating energy demand, or adaptive comfort index) contained missing values, non-physical results, or simulation failures reported by EnergyPlus. In addition, records with clearly non-physical geometric configurations (e.g., zero or negative window dimensions) were removed. After applying these criteria, 1 record was discarded from the original dataset of 9901 samples, resulting in a cleaned dataset of 9900 valid design configurations. No statistical trimming (e.g., percentile-based or z-score–based outlier removal) was applied, in order to preserve the full variability of the design space and avoid biasing the surrogate model toward central regions. The cleaned dataset was subsequently normalized using a StandardScaler and used consistently across surrogate training, validation, and optimization stages.

### Artificial neural network (ANN) model

**Fig. 3:**
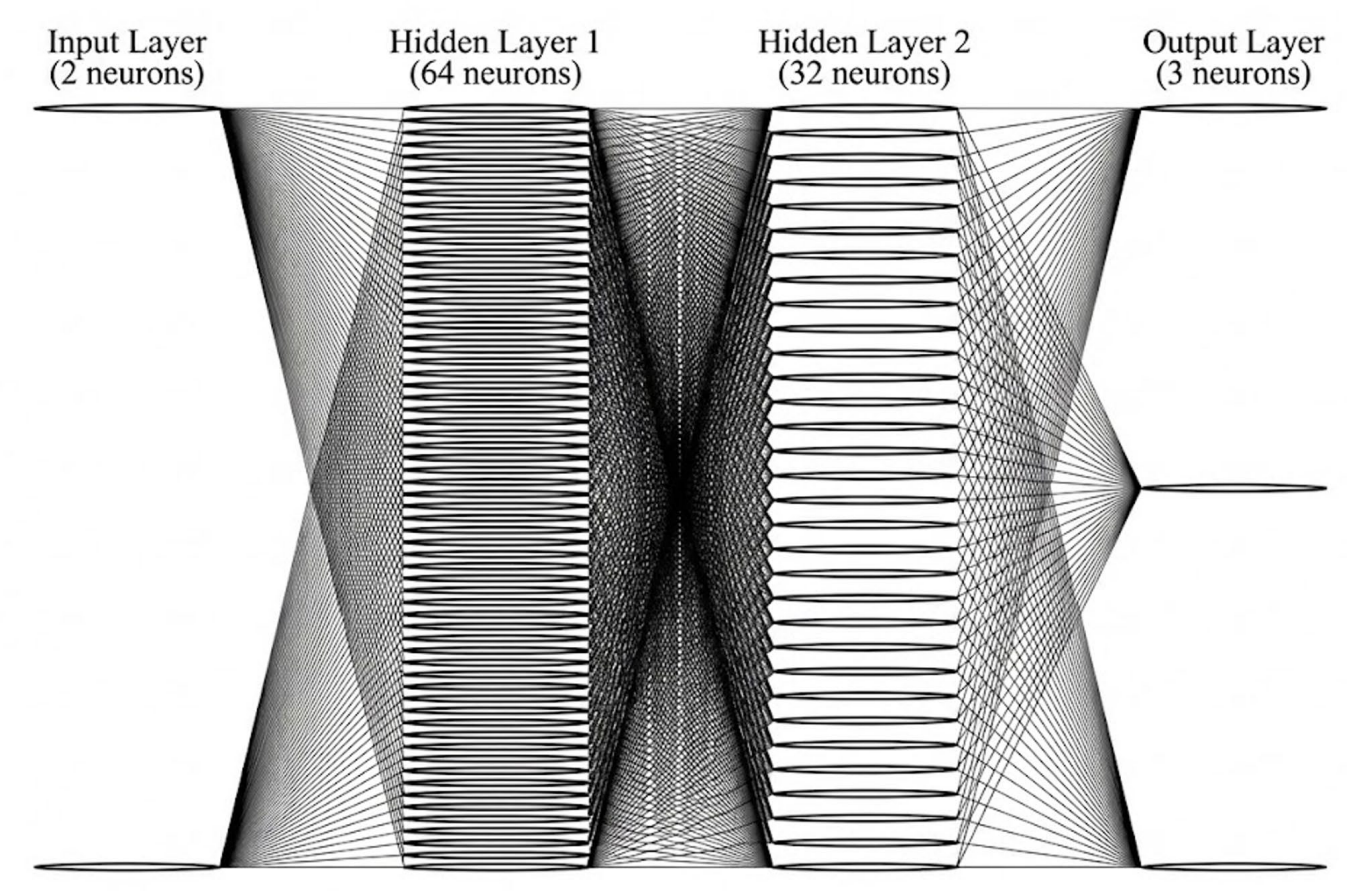
Architecture of the artificial neural network surrogate model used to predict annual cooling energy demand, annual heating energy demand, and adaptive thermal comfort.

An artificial neural network surrogate model was developed using Keras with a TensorFlow backend to predict annual cooling energy demand, annual heating energy demand, and adaptive thermal comfort. The network architecture consists of an input layer corresponding to the transformed geometric inputs, two hidden layers with 64 and 32 neurons respectively using ReLU activation functions, and an output layer with three neurons representing the performance indicators (Fig. 3).

The model was trained using the Adam optimizer and mean squared error (MSE) loss function over 200 epochs with a batch size of 32. The dataset was randomly split into training (80%) and testing (20%) subsets. Model accuracy was evaluated using coefficient of determination (R²) and error-based metrics.

### Multi-objective optimization with NSGA-III

A multi-objective optimization process was conducted using the NSGA-III algorithm implemented through the pymoo library. The optimization objectives were defined as:

- Minimization of annual cooling energy demand,
- Minimization of annual heating energy demand,
- Maximization of adaptive thermal comfort.

The trained ANN surrogate model was employed as a computationally efficient evaluator during the optimization process. The search space was defined over the original window width and height domains. The resulting Pareto-optimal solutions represent trade-offs between energy demand and thermal comfort performance.

Hypervolume evaluation and normalization procedure.

To enable a quantitative comparison between different optimization strategies, the hypervolume (HV) indicator was used as a primary performance metric. Prior to HV calculation, all objective values were min–max normalized to the interval [0, 1] using the minimum and maximum values observed across all algorithms and runs.

Hypervolume was computed with respect to a fixed reference point located at (1.1, 1.1, 1.1) in the normalized objective space, ensuring that all non-dominated solutions contributed positively to the metric. This procedure allows simultaneous assessment of convergence toward the Pareto front and diversity of the obtained solution sets under matched computational budgets.

## Results and discussion

### Surrogate model performance

**Fig. 4:**
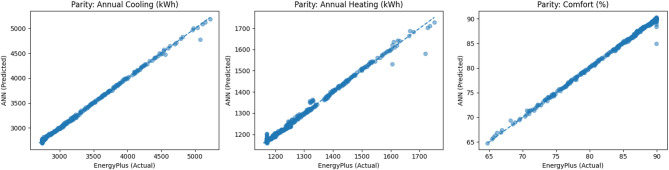
Parity plots comparing the ANN surrogate predictions against EnergyPlus simulation results for annual cooling energy demand, annual heating energy demand, and adaptive thermal comfort index.

The predictive performance of the PCA–ANN surrogate model was evaluated using an independent test dataset comprising 20% of the total samples. Figure 4 presents parity plots comparing ANN predictions against EnergyPlus simulation results for annual cooling energy demand, annual heating energy demand, and the adaptive thermal comfort index. In all three cases, the predicted values closely follow the 45° parity line, indicating strong agreement between the surrogate model and the simulation outputs within the explored design space.

Quantitatively, the surrogate achieved coefficients of determination (R²) of 0.9989 for annual cooling energy demand, 0.9955 for annual heating energy demand, and 0.9982 for the adaptive comfort index. As can be seen in Table 3, these values indicate that the ANN explains the majority of variance in the simulation outputs for this low-dimensional parametric problem. Error-based metrics further confirm the predictive reliability of the model, with mean absolute errors of 5.44 kWh and 2.28 kWh for annual cooling and heating energy demand, respectively. For the adaptive thermal comfort index, the MAE and RMSE were limited to 0.05 and 0.16% points, indicating negligible absolute deviations.

**Fig. 5:**
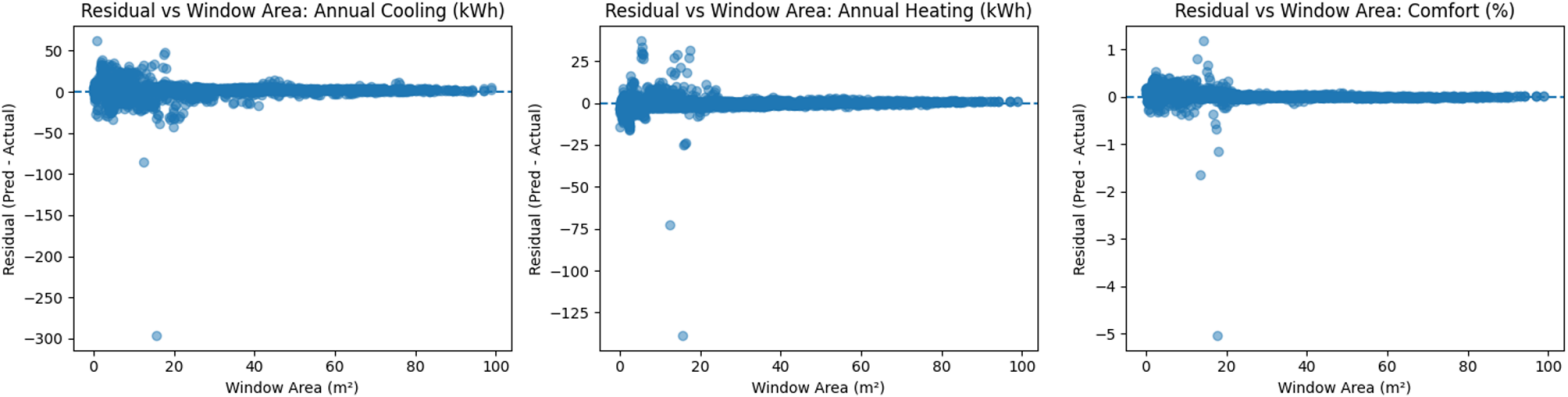
Residual diagnostics of the PCA–ANN surrogate model plotted against window area for (**a**) annual cooling energy demand, (**b**) annual heating energy demand, and (**c**) adaptive thermal comfort index.

To assess whether the high R² values reflect genuine predictive capability rather than overfitting, residual diagnostics were examined across the design space. Figure 5 presents residuals plotted against window area for all three outputs. The residuals are centered around zero and do not exhibit clear systematic trends with increasing window area. While some dispersion is observed for very small window areas, no consistent bias is apparent across the explored range, indicating stable surrogate performance for both small and large window sizes.

In addition, the ANN surrogate was benchmarked against simpler baseline models, including linear regression and second-order polynomial regression (Table 4). The ANN consistently outperformed both baselines across all objectives, achieving substantially higher coefficients of determination. This comparison demonstrates that nonlinear learning is required to capture the relationships between window geometry and building performance, even when the input space is limited to two geometric variables.

Although the input space consists only of window width and height, principal component analysis (PCA) was applied as a preprocessing step to decorrelate and scale the inputs rather than to reduce dimensionality. This preprocessing improved training stability without altering the dimensionality of the design variables. Overall, the results confirm that the PCA–ANN surrogate provides a reliable and computationally efficient approximation of EnergyPlus simulations and is therefore suitable for use as a fast evaluator in the subsequent multi-objective optimization stage.

**Table 3:** Predictive performance of the ANN surrogate model evaluated on the independent test dataset.

Objective	*R*²	MAE	RMSE
Annual Cooling (kWh)	0.9989	5.44 kWh	10.87 kWh
Annual Heating (kWh)	0.9955	2.28 kWh	5.32 kWh
Comfort (%)	0.9982	0.05%	0.16%

To justify the use of a non-linear surrogate, the ANN performance was compared with simple linear and second-order polynomial regression baselines (Table 4). While the ANN achieved R² values above 0.98 for all outputs, both baseline models performed poorly (R² < 0.26), indicating that the relationships between window geometry and performance indicators are strongly non-linear and cannot be captured by simple regression models.

**Table 4:** Comparison of ANN surrogate performance with linear and second-order polynomial regression baselines (R²).

Objective	ANN	Linear	Polynomial (2nd order)
Annual Cooling (kWh)	0.9989	0.12649	0.1973
Annual Heating (kWh)	0.9955	0.16659	0.2520
Comfort (%)	0.9982	0.1572	0.2396

### Multi-objective optimization results

Using the trained PCA–ANN surrogate model as a computationally efficient evaluator, a multi-objective optimization was conducted to explore the trade-offs among annual cooling energy demand, annual heating energy demand, and adaptive thermal comfort. Three optimization strategies were considered: NSGA-II, NSGA-III, and a weighted-sum scalarization baseline.

To ensure a fair and reproducible comparison, all algorithms were executed under identical computational budgets (population size = 100, number of generations = 200) and repeated across ten independent random seeds. Algorithm performance was evaluated using the hypervolume (HV) indicator, computed on min–max normalized objective values with respect to a fixed reference point, to jointly assess convergence and diversity of the resulting solution sets. Total wall-clock runtime and the number of non-dominated solutions were additionally recorded to characterize computational efficiency and solution richness.

Table 5 summarizes the comparative results. For the present three-objective formulation, NSGA-II achieved the highest average hypervolume (1.093 ± 0.003), indicating more effective coverage of the Pareto front and a denser representation of trade-offs. NSGA-III yielded slightly lower hypervolume values (1.049 ± 0.014) and produced a smaller number of Pareto-optimal solutions on average. This behavior is consistent with the design philosophy of NSGA-III, which emphasizes reference-direction–based diversity control and is primarily intended for many-objective optimization problems (typically four or more objectives). When applied to a three-objective problem, this mechanism may limit solution density without providing a corresponding improvement in convergence quality.

In contrast, the weighted-sum baseline consistently converged to a single solution and exhibited substantially lower hypervolume values (0.536 ± 0.000), demonstrating its inability to represent the full trade-off surface inherent in the competing energy–comfort objectives. This result highlights the limitations of scalarization-based approaches for multi-criteria building design optimization.

In terms of computational cost, all three methods exhibited comparable runtimes due to the use of the ANN surrogate model, confirming that surrogate-assisted optimization enables rapid exploration of the design space and is suitable for early-stage design decision support. Overall, the results indicate that while NSGA-III remains a robust and scalable optimizer for higher-dimensional objective spaces, NSGA-II provides superior performance and richer Pareto fronts for the three-objective window optimization problem considered here. The inclusion of NSGA-III therefore serves as a methodological benchmark and as a forward-compatible choice for future extensions involving additional objectives. Accordingly, NSGA-III is not claimed to be necessary or superior for the present three-objective formulation. Its inclusion is intended to (i) provide a methodological benchmark against NSGA-II and a scalarization baseline, and (ii) demonstrate the extensibility of the proposed framework toward future studies involving higher-dimensional objective spaces. For the specific problem considered here, NSGA-II offers a more effective balance between convergence and solution diversity.

**Table 5:** Performance comparison of NSGA-II, NSGA-III, and weighted-sum optimization approaches based on hypervolume, runtime, and number of Pareto-optimal solutions (mean ± standard deviation across ten runs).

Algorithm	Hypervolume (mean ± SD)	Runtime (s) (mean ± SD)	Number of Pareto Solutions
NSGA-II	1.093 ± 0.003	32.0 ± 0.5	100
NSGA-III	1.049 ± 0.014	32.5 ± 0.6	27
Weighted-sum	0.536 ± 0.000	31.9 ± 0.6	1

### Design space analysis

Analysis of the Pareto-optimal solutions in the decision-variable space (window width and height) provides further insight into the mechanisms underlying the observed objective-space trade-offs. Because the optimization varies only window geometry while maintaining fixed orientation, glazing properties, and envelope characteristics, multiple width–height combinations yield comparable performance when the resulting window area is similar. This confirms that, within the studied bounds, window area rather than aspect ratio is the dominant geometric driver of performance in the simplified shoebox model.

Across the explored design space, annual cooling energy demand increases almost monotonically with increasing window area. This trend reflects the proportional relationship between south-facing glazing area and solar heat gains during the cooling season in a hot-dry climate. In contrast, annual heating energy demand decreases with increasing window area, indicating that passive winter solar gains outweigh additional conductive heat losses under the assumed envelope and operational conditions.

The adaptive thermal comfort index also increases with window area within the investigated range. This trend must be interpreted in the context of the adopted comfort definition and simulation scenario. Comfort is quantified as the percentage of occupied hours within the ASHRAE 55 adaptive comfort range under free-running conditions. This metric does not explicitly account for the magnitude or duration of overheating events (e.g., degree-hours above upper comfort thresholds). Consequently, although larger windows may plausibly increase summertime overheating risk, such effects are not fully captured by the selected comfort indicator. The monotonic increase in comfort with window size should therefore not be interpreted as evidence that larger windows universally improve summer thermal conditions.

**Fig. 6:**
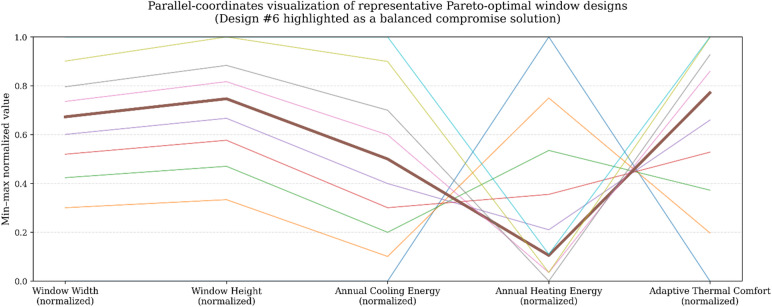
Parallel-coordinates visualization of representative Pareto-optimal window designs. All variables are min–max normalized to the interval [0,1] for visualization purposes only. Window width and height denote normalized geometric parameters; while cooling and heating correspond to annual energy demand (kWh), and comfort represents adaptive thermal comfort coverage (%). The figure illustrates relative trade-off patterns rather than absolute values; exact numerical results are reported in Table 6. Design #6 is highlighted as a balanced compromise between energy demand and thermal comfort.

Overall, the design-space analysis confirms that window area is the primary determinant of thermal and comfort performance in this simplified early-stage model. The Pareto-optimal solutions provide a transparent basis for selecting window dimensions according to project priorities, explicitly revealing the trade-offs between cooling demand, heating demand, and adaptive comfort. Representative Pareto-optimal window configurations and their predicted performance are reported in Table 6; Fig. 6.

**Table 6:** Representative Pareto-optimal window designs and their predicted performance outcomes.

Design	Window width (m)	Window height (m)	Annual cooling energy (kWh)	Annual heating energy (kWh))	Comfort Index (%)
1	1.00	1.00	556	1150	58.1
2	1.41	1.41	611	1107	65.3
3	1.73	1.73	667	1071	71.7
4	2.00	2.00	722	1042	77.1
5	2.24	2.24	778	1021	81.7
6	2.45	2.45	833	1007	85.3
7	2.65	2.65	889	1000	88.1
8	3.00	3.00	1000	1007	91.0
9	3.33	3.00	1056	1022	91.1

### Comparison with previous studies

The trends observed in this study are consistent with previous research on window and façade optimization in warm and hot-dry climates. Numerous studies have reported that increasing glazing area can reduce heating demand through enhanced passive solar gains while simultaneously increasing cooling demand due to higher solar heat gains in summer. Similarly, comfort-related indicators often improve with increased daylight and solar access, although this improvement depends strongly on the selected comfort model, climate, and operational assumptions.

From a methodological perspective, the present work aligns with a growing body of literature employing surrogate-assisted optimization to reduce the computational burden of simulation-driven design exploration. In this context, the ANN surrogate enables rapid evaluation of candidate solutions, while the evolutionary optimization framework facilitates the identification of trade-offs among competing objectives. Importantly, the present study does not claim algorithmic superiority of NSGA-III over other evolutionary methods for three-objective problems. Rather, NSGA-III is employed as a robust and extensible optimizer capable of generating a diverse set of Pareto-optimal solutions, with explicit quantitative comparisons provided to benchmark its performance against alternative strategies.

By focusing on a minimal and controlled design problem, this study complements prior work on more complex geometries and larger design spaces, providing a transparent reference case for understanding how window size alone influences annual energy demand and adaptive comfort in early-stage design.

## Conclusion

This study presented an AI-driven multi-objective framework for optimizing window dimensions in a simplified one-story office shoebox model representative of Tehran’s hot-dry climate. Using parametric simulations with Ladybug Tools and Honeybee coupled to EnergyPlus, a comprehensive dataset was generated by systematically varying window width and height. For each configuration, annual cooling energy demand (kWh), annual heating energy demand (kWh), and an adaptive thermal comfort index—defined as the percentage of occupied hours within the ASHRAE 55 adaptive comfort range—were evaluated.

A PCA–ANN surrogate model trained on decorrelated geometric inputs demonstrated high predictive accuracy across all outputs, with coefficients of determination exceeding 0.99 and low absolute prediction errors. These results indicate that the surrogate model provides a reliable and computationally efficient approximation of EnergyPlus simulations, enabling rapid performance evaluation within a simulation-based optimization workflow. When coupled with multi-objective evolutionary optimization, the framework produced a well-defined Pareto set that explicitly captures the trade-offs between cooling energy demand, heating energy demand, and adaptive thermal comfort.

The optimization results indicate that increasing window area generally reduces annual heating energy demand and improves adaptive comfort coverage, while simultaneously increasing annual cooling energy demand. This trade-off must be interpreted in the context of the local climatic balance between heating and cooling seasons. Based on the Tehran weather file used in this study, the heating-dominated period extends over approximately seven months of the year, while the cooling season is comparatively shorter, spanning roughly four to five months. This seasonal asymmetry partly explains why moderate increases in window area can yield net annual energy benefits despite higher summer cooling loads.

This behavior reflects the expected energy–comfort trade-off in hot-dry climates and highlights the importance of balanced window sizing during early-stage design. Rather than identifying a single optimal solution, the Pareto-optimal set provides designers with a spectrum of high-performance alternatives that can be selected according to project-specific priorities. For example, window dimensions around 2.2–2.5 m (square configuration) represent a balanced compromise in the present case study, achieving moderate cooling energy demand while substantially reducing heating demand and maintaining adaptive comfort levels above 80%. This illustrates how intermediate window sizes can effectively balance the opposing seasonal energy requirements in hot-dry climates.

The main limitations of this study arise from its simplified modeling assumptions, including the use of a single-zone shoebox geometry, fixed envelope properties and orientation, and reliance on a single adaptive comfort indicator evaluated under free-running conditions. As a result, overheating severity under extreme summer conditions may not be fully captured, and the reported energy–comfort relationships should be interpreted in a comparative rather than absolute sense. In addition, surrogate model validation was performed against EnergyPlus simulations rather than field measurements, which limits direct real-world generalization.

Future research will extend the proposed framework to more complex and realistic building configurations, including multi-zone and multi-story models, and will incorporate additional design variables such as glazing properties, shading devices, orientation, and ventilation strategies. The inclusion of overheating-based discomfort metrics and alternative comfort models will further enhance the external validity and practical relevance of the framework, supporting more robust performance-informed decision-making in real-world building design.

## Data Availability

Reproducibility materials supporting this study have been publicly archived and are available at Zenodo: [**https://doi.org/10.5281/zenodo.18456422**](https:/doi.org/10.5281/zenodo.18456422) .The repository includes:- cleaned parametric simulation data (data2\_cleaned.csv),- scripts for PCA preprocessing and surrogate training,- baseline regression scripts,- optimization benchmarking scripts (NSGA-II, NSGA-III, weighted-sum),- associated post-processing and visualization scripts,- a README file specifying exact software versions and execution instructions to reproduce the reported results.These materials ensure full transparency and reproducibility of the modeling and optimization workflows presented in this paper.
